# Plasma endotrophin levels correlate with insulin resistance in people with obesity

**DOI:** 10.1172/JCI190577

**Published:** 2025-04-22

**Authors:** Gordon I. Smith, Samuel Klein

**Affiliations:** Center for Human Nutrition, Washington University School of Medicine, St. Louis, Missouri, USA.

**Keywords:** Clinical Research, Metabolism, Muscle biology, Adipose tissue, Extracellular matrix, Insulin

**To the Editor:** The increase in adipose tissue mass associated with obesity requires extracellular matrix (ECM) remodeling to provide the scaffolding needed to support tissue expansion. However, excessive ECM formation and subsequent fibrosis are associated with insulin-resistant glucose metabolism, which is an important factor in the pathogenesis of obesity-related cardiometabolic diseases (1). The mechanism responsible for the link between adipose tissue fibrosis and systemic insulin resistance is unclear but could involve an increase in plasma endotrophin, a peptide cleaved from the α3 chain of collagen VI during fibrogenesis, because adipose tissue–derived endotrophin causes systemic insulin resistance in rodents (2, 3), and high plasma endotrophin is associated with increased cardiovascular disease risk (4) and mediates the link between obesity and cardiovascular disease in people (5). Here, we present results from a series of studies that evaluated the potential importance of circulating endotrophin in regulating insulin action in people.

In study 1, plasma endotrophin concentrations were assessed in people who were lean and insulin sensitive (lean-IS; *n* = 10), people who were obese and insulin sensitive (obese-IS; *n* = 10), and people who were obese and insulin resistant (obese-IR; *n* = 10). The lean-IS and obese-IS groups were matched by insulin sensitivity, assessed by the hyperinsulinemic-euglycemic clamp procedure, and the obese-IS and obese-IR groups were matched by sex, BMI, and percentage body weight as fat (Supplemental Table 1; supplemental material available online with this article; https://doi.org/10.1172/JCI190577DS1). Fasting plasma endotrophin concentrations were approximately 30% higher in the obese-IR group than the lean-IS and obese-IS groups (Figure 1A). The effect of insulin sensitivity and the ingestion of mixed meals on serial 24-hour plasma endotrophin concentrations were assessed in 4 of the most insulin-sensitive and 4 of the most insulin-resistant participants, matched by BMI (42.0 ± 3.0 vs. 42.7 ± 2.4 kg/m^2^, respectively). The serial 24-hour plasma endotrophin AUC was approximately 25% greater in the obese-IR group than the obese-IS group, with minimal variability in plasma endotrophin concentrations, despite marked meal-induced increases in plasma glucose and insulin concentrations (Figure 1, B–D). The stability of plasma endotrophin throughout the day demonstrates that basal plasma endotrophin concentration is a reasonable indicator of total daily circulating levels and is consistent with a previous study that showed *COL6A3* expression in human adipose tissue explant cultures is not affected by acute hyperglycemia or hyperinsulinemia (6). Fasting plasma endotrophin concentrations were also associated with measures of glucose homeostasis and insulin sensitivity; plasma endotrophin was directly correlated with 24-hour plasma glucose and insulin AUCs and with intrahepatic triglyceride (IHTG) content, and was inversely correlated with hepatic and whole-body insulin sensitivity (Supplemental Figure 1, A–E). The correlation between whole-body insulin sensitivity and the plasma endotrophin/adiponectin ratio was the same as for plasma endotrophin alone, and both correlations were greater than the correlation between whole-body insulin sensitivity and the plasma leptin/adiponectin ratio (Supplemental Figure 1, E–G). The cellular factors in adipose tissue associated with endotrophin production (markers of hypoxia, inflammation, fibrosis, and matrix metalloproteinases) were evaluated by RNA-Seq. Expression of these factors progressively increased from the lean-IS to the obese-IS to the obese-IR group and were positively associated with plasma endotrophin concentrations (Supplemental Figure 2).

In study 2, we assessed plasma endotrophin concentrations in 15 people with obesity and type 2 diabetes (52 ± 2 years old) before and after marked weight loss (18.3% ± 0.6%; range, 15.7%–24.7%) induced by Roux-en-Y gastric bypass (RYGB) surgery (*n* = 6) or intensive diet therapy (*n* = 9). Weight loss increased whole-body insulin sensitivity by approximately 150% and decreased IHTG content and hemoglobin A1c) (Supplemental Table 2). Weight loss decreased plasma endotrophin concentration by approximately 15% (*P* = 0.006; Figure 1E), without a difference in the decrease in the plasma endotrophin concentration between the RYGB surgery and intensive diet therapy groups (2-way ANOVA group × time interaction; *P* = 0.48).

In study 3, primary human skeletal muscle myotubes were incubated with endotrophin to determine whether endotrophin directly affects insulin action. Endotrophin treatment completely inhibited insulin-stimulated glucose uptake (Figure 1F). Adding a neutralizing endotrophin antibody to endotrophin-treated myotubes restored insulin-stimulated glucose uptake (Figure 1F).

These studies support the idea that plasma endotrophin is a signaling mechanism that links adipose tissue fibrogenesis with systemic insulin resistance in people with obesity. Our findings demonstrate that plasma endotrophin tracks with whole-body insulin sensitivity, is higher in obese-IR than obese-IS, and decreases in conjunction with a weight loss–induced improvement in insulin action. Moreover, endotrophin impairs insulin-stimulated glucose uptake in human skeletal muscle myotubes, demonstrating a direct effect on insulin action. These results suggest that in people with obesity and insulin resistance, plasma endotrophin is both a biomarker and cause of whole-body insulin resistance and is a potential therapeutic target in these individuals.

Additional details on methods are available in the supplemental materials.

## Supplementary Material

Supplemental data

ICMJE disclosure forms

Supporting data values

## Figures and Tables

**Figure 1:**
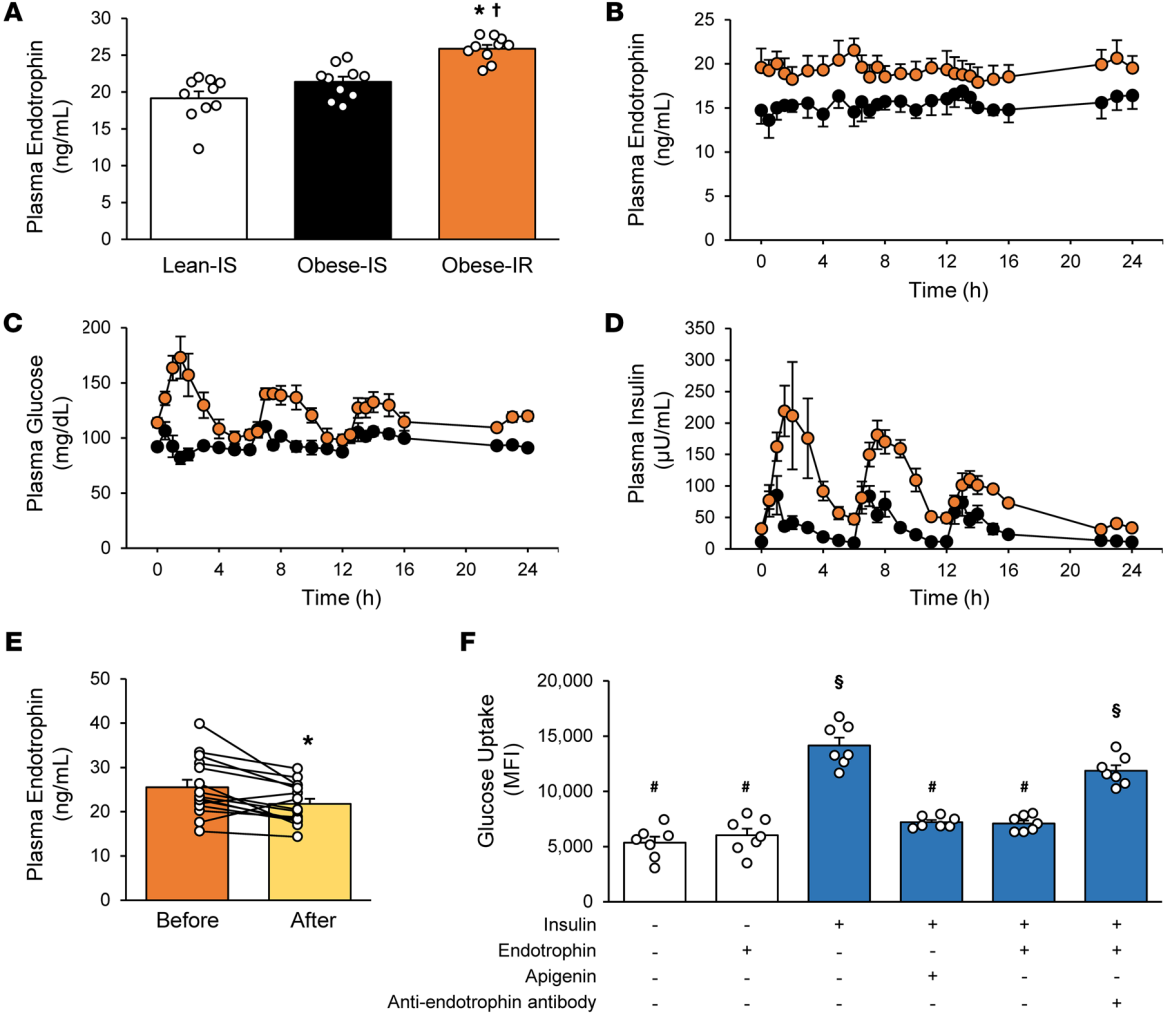
Fasting plasma endotrophin, 24-hour plasma endotrophin, glucose and insulin profiles, and effect of endotrophin on insulin action. (**A**) Basal plasma endotrophin concentrations in lean-IS (*n* = 10), obese-IS (*n* = 10) and obese-IR (*n* = 10), groups. *P < 0.05 versus lean-IS and ^†^*P* < 0.05 versus obese-IS, by Tukey’s honest significant difference (HSD) test. (**B**–**D**) Serial plasma endotrophin (**B**), glucose (**C**), and insulin (**D**) concentrations assessed over 24 hours with meals provided at 0 (0700 hours), 6 (1300 hours), and 12 (1900 hours) hours in a subset of participants in the obese-IS (*n* = 4; black) and obese-IR (*n* = 4; orange) groups. (**E**) Plasma endotrophin concentrations before and after approximately 18% weight loss in people with obesity and type 2 diabetes (*n* = 15). *P < 0.05 versus before, by 2-tailed Students *t* test for paired samples. (**F**) Glucose uptake in myotubes treated with and without insulin, apigenin (an inhibitor of glucose uptake), endotrophin, and/or a neutralizing anti-endotrophin monoclonal antibody. Conditions with different symbols are significantly different (*P* < 0.01) from each other by Games-Howell test. Data are the mean ± SEM. Individual values are shown as open circles in **A**, **E**, and **F**.

## Version 1 04/22/2025

In-Press Preview

## Version 2 06/16/2025

Electronic publication
