# Supplementary material for: Plasma endotrophin levels correlate with insulin resistance in people with obesity
Source: J Clin Invest. 2025 Apr 22;135(12):e190577. doi: 10.1172/JCI190577 (PMC12165808; doi:10.1172/JCI190577)
Supplement: Supplemental data [file jci-135-190577-s207.pdf]

## SUPPLEMENTAL METHODS

### Sex as a biologic variable.

Our study is unable to assess the effect of sex because the Obese-IS and Obese-IR groups did not include males because it is difficult to find males with obesity that are insulin sensitive. The Lean-IS group included 5 men and 5 women. In the weight loss study, more women (n=10) participated than men (n=5). As a result, we are underpowered to determine whether responses differed between men and women in this study.

### Study Participants

The samples analyzed in Studies 1 and 2 were obtained from a subset of participants as part of their participation in two other studies (1, 2). Both studies were approved by the Human Research Protection Office at Washington University School of Medicine in St. Louis, MO and registered in ClinicalTrials.gov (NCT02706262 and NCT02207777). Written, informed consent was obtained from all participants before they started the study, which were conducted in the Clinical Translational Research Unit (CTRU) at Washington University School of Medicine in St. Louis, MO.

Study 1 involved 30 men and women who were Lean-IS (n=10; 5 men and 5 women; 5 White, 1 Black, and 4 Asian people), Obese-IS (n=10; all women; 6 White and 4 Black people) or Obese-IR (n=10; all women; 7 White and 2 Black people, and 1 person from the Pacific Islands), and was conducted from May 2016 to August 2019. The following inclusion criteria were required for each cohort: i) Lean-IS defined as BMI 18.5-24.9 kg/m$^2$, fasting plasma glucose concentration <100 mg/dL, 2-hr oral glucose tolerance test (OGTT) plasma glucose concentration ≤140 mg/dL, HbA1c ≤5.6% and normal whole-body insulin sensitivity, defined

as the glucose infusion rate (GIR) per kg fat-free mass (FFM) divided by the plasma insulin concentration (GIR/Insulin) during the final 20 minutes of the hyperinsulinemic-euglycemic clamp procedure >40 (μg/kg FFM/min)/(μU/mL); ii) Obese-IS had BMI 30-49.9 kg/m$^2$, fasting plasma glucose concentration <100 mg/dL, 2-hr OGTT plasma glucose concentration ≤140 mg/dL, HbA1c ≤5.6% and normal whole-body insulin sensitivity; and iii) Obese-IR had BMI 30-49.9 kg/m$^2$, HbA1c 5.7%-6.4% or fasting plasma glucose concentration 100-125 mg/dL or 2-hr OGTT plasma glucose concentration 140-199 mg/dL and impaired whole-body insulin sensitivity, defined as a GIR/Insulin ≤40 (μg/kg FFM/min)/(μU/mL). Potential participants who had a history of diabetes or liver disease other than metabolic dysfunction-associated steatotic liver disease (MASLD), were taking medications that can affect metabolism, had metal implants that precluded magnetic resonance imaging or consumed excessive amounts of alcohol (>21 units of alcohol per week for men and >14 units of alcohol per week for women) were excluded.

Study 2 involved 15 people with obesity and type 2 diabetes (T2D) (age 52±2 years; 5 men and 10 women; 11 White and 3 Black people and 1 Asian person), and was conducted from November 2014 to April 2018. Participants were included if they achieved marked (>15%) weight loss, induced by either Roux-en-Y gastric bypass surgery (n=6) or low-calorie diet therapy (n=9). This weight loss target was chosen because it is associated with a high rate of remission of T2D (3). Potential participants who had evidence of significant organ system dysfunction or disease other than MASLD and T2D, previous intestinal resection, or consumed excessive amounts of alcohol (>21 oz of alcohol per week for men and >14 oz of alcohol per week for women) were excluded.

**Body composition analyses**

Body fat mass and FFM were determined by using dual-energy x-ray absorptiometry (Lunar iDXA; GE, Madison, WI) and IHTG content was determined by using magnetic resonance imaging-proton density fat fraction (analyzed by AMRA Medical AB, Linköping, Sweden) in Study 1 and by magnetic resonance spectroscopy as previously described (4) in Study 2.

**Serial 24-h blood sampling and hyperinsulinemic-euglycemic clamp procedure in Study 1**

Participants were admitted to the CTRU at 1700 h on day 1 for ~48 hours. Subjects were given standard meals (50% carbohydrate, 35% fat, 15% protein), each containing one-third of their estimated energy requirements (5), at 1900 h on the day of admission and at 0700 h, 1300 h, and 1900 h on day 2. At 0630 h on day 2, a catheter was inserted into a forearm or antecubital vein for 24-h serial blood sampling. Blood samples were obtained hourly from 0700 h to 2300 h on day 2 and from 0500 h to 0700 h on day 3 with additional blood samples obtained at 30 and 90 min after each meal. At 0715 h on day 3, a primed (8.0 µmol/kg) continuous (0.08 µmol/kg/min) infusion of [U-$^{13}$C]glucose (Cambridge Isotope Laboratories Inc., Andover, MA) was started through the existing intravenous catheter. An additional catheter was inserted into a radial artery to obtain arterial blood samples. After the infusion of glucose tracer for 210 min (basal period), insulin was infused for 210 min at a rate of 50 mU/m$^2$ body surface area (BSA)/min (initiated with a two-step priming dose of 200 mU/m$^2$ BSA/min for 5 min followed by 100 mU/m$^2$ BSA/min for 5 min). The infusion of [U-$^{13}$C]glucose was stopped during insulin infusion because of the expected decrease in hepatic glucose production (6). Euglycemia (~100 mg/dl) was maintained by variable infusion

of 20% dextrose enriched to ~1% with [U-$^{13}$C]glucose. Blood samples were obtained before beginning the tracer infusion to assess plasma endotrophin concentration and every 6-7 min during the final 20 min (total of 4 blood samples) of the basal period to assess hepatic insulin sensitivity and during the final 20 min of insulin infusion to assess whole-body insulin sensitivity.

**Pancreatic hyperinsulinemic-euglycemic clamp procedure in Study 2**

To assess insulin sensitivity, participants completed a 9-hour, three-stage pancreatic hyperinsulinemic-euglycemic clamp procedure, as described previously (1). Glucagon-like peptide-1 receptor agonists were discontinued two weeks before, oral diabetes medications were discontinued three days before, and insulin was discontinued one day before each metabolic study to eliminate their impact on insulin action. Participants were admitted to the CTRU in the afternoon before the clamp procedure where they consumed a standard dinner and then fasted overnight. The following morning, catheters were inserted into an arm vein to infuse stable isotopically labeled glucose, 20% dextrose, insulin, octreotide, glucagon, and growth hormone, and into a radial artery for blood sampling. Blood samples were obtained immediately before starting the clamp procedure to assess fasting plasma endotrophin concentration and during the last 20 minutes of the high insulin infusion stage (50 mU insulin/m$^2$ body surface area/min) to assess whole-body insulin sensitivity.

**Adipose tissue biopsies**

In Study 1, subcutaneous abdominal adipose tissue (SAAT) was obtained from the periumbilical area during the basal period of the clamp procedure. After anesthetizing the skin by percutaneous injection of 1% lidocaine, a small skin incision (~0.5 cm) was made and ~0.5 grams of adipose tissue was aspirated through a 4-mm liposuction cannula (Tulip Medical Products, San Diego, CA) connected to a 60 cc syringe. These samples were immediately rinsed in ice-cold saline and frozen in liquid nitrogen before being stored at -80°C until processed for RNA sequencing.

**Insulin-stimulated glucose uptake in human skeletal muscle myotubes**

Studies conducted in rodents have shown endotrophin affects whole-body insulin sensitivity (7, 8). We therefore chose to examine the effect of endotrophin on insulin-stimulated glucose uptake in primary human skeletal muscle myotubes because skeletal muscle is the primary determinant of whole-body insulin stimulated glucose uptake (9-11).

Human Skeletal Muscle Myoblasts (HSMM cells; Lonza, CC-2580) were cultured in skeletal muscle growth medium and incubated under 5% $CO_2$ at 37$^{o}$ C. After reaching 50% confluence, the growth medium was removed and replaced with an equal volume of fusion medium (DMEM-F12 supplemented with 2% horse serum). Cells were cultured until multinucleated myotubes were detected at which point fusion medium was removed and horse serum-free skeletal muscle growth medium added. Glucose uptake was assessed by using a cell-based assay kit from Cayman Chemical (Cat # 600470). A 96-well plate was seeded with $5X10^4$ HSMM cells/well in 100 µl culture medium. Cells were grown overnight. The next day, cells were treated with endotrophin at 1 µg/ml, with endotrophin exposed to a neutralizing antibody (5 µg/ml), or with the insulin receptor inhibitor apigenin (a 1:500 dilution from 50

mM stock), and insulin was added to some cells at 100 nM for 20 min in glucose-free culture medium. Ten minutes before the end of the treatment, 2-NBDG was added to a final concentration of 200 µg/ml in glucose-free medium. At the end of the treatment, the plate was centrifuged for 5 min. The supernatant was aspirated, 200 µl of cell-based assay buffer was added, centrifuged again, and the supernatant aspirated. Another 100 µl of cell-based assay buffer was added, and the cells were analyzed. 2-NBDG uptake by cells was detected by fluorescence (excitation/emission=485/535 nm).

**Sample analyses and calculations**

*Plasma sample analysis and concentrations.* Plasma glucose concentration was determined by using an automated glucose analyzer (Yellow Spring Instruments Co, Yellow Springs, OH). Plasma insulin and HbA1c were measured in the Washington University Core Laboratory for Clinical Studies. Plasma endotrophin (12), adiponectin, and leptin (both MilliporeSigma, Burlington, MA) concentrations were determined by using ELISAs. Plasma endotrophin, glucose and insulin concentration 24-h AUC were calculated using the trapezoidal method (13). Plasma glucose tracer-to-tracee ratio was determined by using gas chromatography-mass spectrometry (14).. The hepatic insulin sensitivity index (HISI) was calculated as the inverse of the product of plasma insulin concentration and the endogenous glucose rate of appearance (Ra) into the systemic circulation, determined by dividing the glucose tracer infusion rate by the average plasma glucose TTR during the last 20 min of the basal period of the HECP (15). Total glucose rate of disappearance (Rd) during insulin infusion was assumed to be equal to the sum of endogenous glucose rate of appearance into the bloodstream and the rate of infused glucose during the last 20 min of the clamp procedure.

Whole-body insulin sensitivity was calculated as glucose Rd expressed per kg FFM divided by the average plasma insulin concentration (glucose $R_d$/I) during the final 20 min of the clamp procedure (15).

*Adipose tissue RNA sequencing.* Total RNA was isolated from frozen SAAT samples by using QIAzol lysis reagent and a RNeasy mini kit (Qiagen, Valencia, CA) in combination with a RNase-free DNase Set (Qiagen) as we previously described (16). Library preparation on the remaining samples was performed with total RNA and cDNA fragments sequenced on an Illumina NovaSeq 6000 (Illumina, San Diego, CA). Gene expression was initially calculated as $\log_2$-transformed counts per million (CPM) reads before being transformed to Z-scores calculated by normalizing values using the mean and standard deviation across all participants. This normalization method allows for direct comparison of genes with varying expression levels, where values of +1 or -1 indicate expression levels one standard deviation above or below the mean, respectively. Composite collagen expression was calculated as the mean Z-score of collagens *1A1*, *1A2*, *3A1*, *5A1*, *5A2*, *5A3*, *6A1*, *6A2*, *6A3*, *12A1*, *14A1* and *24A1*, which we have found to be closely related to adipose tissue fibrogenesis (collagen 1α1 fractional synthesis rates) and expression of genes that regulate extracellular matrix formation (17).

**Statistics**

All non-RNA sequencing data were tested for homogeneity of variance by Levene's test and normality by using the Kolmogorov-Smirnov test with skewed data sets were log-transformed for inferential statistical analysis and back transformed for presentation. One-way ANOVA was used to compare participant characteristics and outcomes measured at a

single time point among Lean-IS, Obese-IS and Obese-IR groups and glucose uptake assessed in human primary skeletal muscle myotubes with the Tukey's honestly significant difference or Games-Howell post-hoc procedures used to identify significant mean differences where appropriate for datasets with equal and unequal variance, respectively. Plasma glucose, insulin and endotrophin 24-h profiles were analyzed by using linear mixed model analysis with time and group as fixed factors. Relationships among plasma endotrophin and other outcomes were evaluated by using linear and nonlinear regression analysis with the best fit to the data reported. Student's t-tests for paired samples were used to compare outcomes before and after weight loss in Study 2. A two-tailed *P* value <0.05 was considered statistically significant. Data are reported as means ± SEM unless otherwise noted. Statistical analyses were conducted by using SPSS (version 28, IBM, Armonk, NY).

**Study approval**

Subjects provided written, informed consent before participating in these studies, which were approved by the Human Research Protection Office at Washington University School of Medicine in St. Louis.

**Data availability**

The RNA-sequencing data have been deposited in the Gene Expression Omnibus database (https://www.ncbi.nlm.nih.gov/geo; accession no.:GSE244121). Raw data used to

generate the descriptive statistics presented in this manuscript can be found in the Supporting Data Values file.

**ACKNOWLEDGEMENTS**

The authors thank Drs. Dawei Bu, Zhiqiang An and Ningyan Zhang for analysis of plasma endotrophin concentrations, the staff of the Center for Human Nutrition, the Clinical and Translational Research Unit and the Center for Clinical Imaging Research for their help in performing this study, and the study subjects for their participation. This study was supported by National Institutes of Health grant P30DK56341 (Nutrition Obesity Research Center), and support from the Foundation for Barnes-Jewish Hospital.

**AUTHOR CONTRIBUTIONS**

G.I.S. conducted the clinical studies. G.I.S. performed the sample analysis. G.I.S, and S.K. analyzed and interpreted the data. G.I.S. and S.K. wrote the manuscript. S.K. designed and supervised the studies and obtained funding for the work. S.K. is the guarantor of this work and, as such, had full access to all the data in the study and takes responsibility for the integrity of the data and the accuracy of the data analysis. All authors critically reviewed and edited the manuscript.

**Supplemental Table 1.** Body composition and metabolic outcomes

| | Lean-IS (n=10) | Obese-IS (n=10) | Obese-IR (n=10) |
|---|---|---|---|
| Age (yr) | 35.1 ± 3.4 | 39.5 ± 2.5 | 39.2 ± 2.0 |
| Body mass index (kg/m$^2$) | 22.8 ± 0.5 | 38.9 ± 1.6[a] | 38.6 ± 1.7[a] |
| Body mass (kg) | 62.8 ± 2.0 | 108.0 ± 6.4[a] | 107.0 ± 4.3[a] |
| Fat-free mass (kg) | 45.2 ± 2.2 | 51.6 ± 2.4[a] | 54.7 ± 1.6[a] |
| Body fat (%) | 28 ± 2 | 51 ± 2[a] | 48 ± 1[a] |
| IHTG content (%) | 1.7 ± 0.2 | 2.6 ± 0.2 | 16.5 ± 2.0[ab] |
| HbA1c (%) | 5.0 ± 0.1 | 5.1 ± 0.1 | 5.8 ± 0.3[ab] |
| Fasting glucose (mg/dL) | 84 ± 1 | 88 ± 1 | 97 ± 3[a] |
| OGTT 2-h plasma glucose (mg/dL) | 92 ± 6 | 104 ± 6 | 176 ± 10[ab] |
| 24-h glucose area under the curve (mg/dL x 24h) | 2,286 ± 63 | 2,268 ± 47 | 2,660 ± 51[ab] |
| 24-h insulin area under the curve (µU/mL x 24h) | 585 ± 111 | 840 ± 73[a] | 2,090 ± 348[ab] |
| Plasma leptin-adiponectin ratio: (ng/mL) / (µg/mL) | 1.5 ± 0.5 | 14.5 ± 3.5[a] | 18.2 ± 3.0[a] |
| Plasma endotrophin-adiponectin ratio: (µg/mL) / (ng/mL) | 1.8 ± 0.4 | 2.1 ± 0.3 | 4.5 ± 0.5[ab] |
| Hepatic insulin sensitivity index: 1,000/((µmol/kg FFM/min) x (µU/mL)) | 11.5 ± 1.6 | 6.7 ± 0.7[a] | 3.0 ± 0.4[ab] |
| Insulin sensitivity: Glucose Rd (nmol/kg FFM/min) / Insulin (µU/mL) | 554 ± 40 | 536 ± 53 | 202 ± 10[ab] |

Data are expressed as mean ± SEM. FFM, fat-free mass. Glucose Rd, glucose rate of disappearance. IHTG, intrahepatic triglyceride. Two-tailed one-way ANOVAs and Tukey's honestly significant difference post-hoc procedures were used to identify significant differences between groups for data with equal variance and Games-Howell post-hoc test for data with unequal variance. [a]*P* ≤0.05 value significantly different from Lean-IS value. [b]*P* ≤0.05 value significantly different from Obese-IS value.

**Supplemental Table 2.** Body composition and metabolic outcomes before and after ~18% weight loss

| | Before | After | P-value |
|---|---|---|---|
| Body mass index ($kg/m^2$) | 42.4 ± 1.4 | 34.6 ± 1.1 | - |
| Body mass (kg) | 118.3 ± 4.1 | 96.4 ± 2.9 | - |
| Fat-free mass (kg) | 60.7 ± 3.0 | 55.5 ± 2.6 | <0.001 |
| Body fat (%) | 48.3 ± 1.7 | 42.6 ± 1.9 | <0.001 |
| IHTG content (%) | 14.7 ± 2.8 | 3.8 ± 0.4 | 0.001 |
| HbA1c (%) | 7.8 ± 0.5 | 5.7 ± 0.2 | <0.001 |
| Insulin sensitivity: Glucose Rd (nmol/kg FFM/min) / Insulin (μU/mL) | 262 ± 30 | 552 ± 45 | <0.001 |

Data are expressed as mean ± SEM. P-values by paired two-tailed t-test. Glucose Rd, glucose rate of disappearance. IHTG, intrahepatic triglyceride.

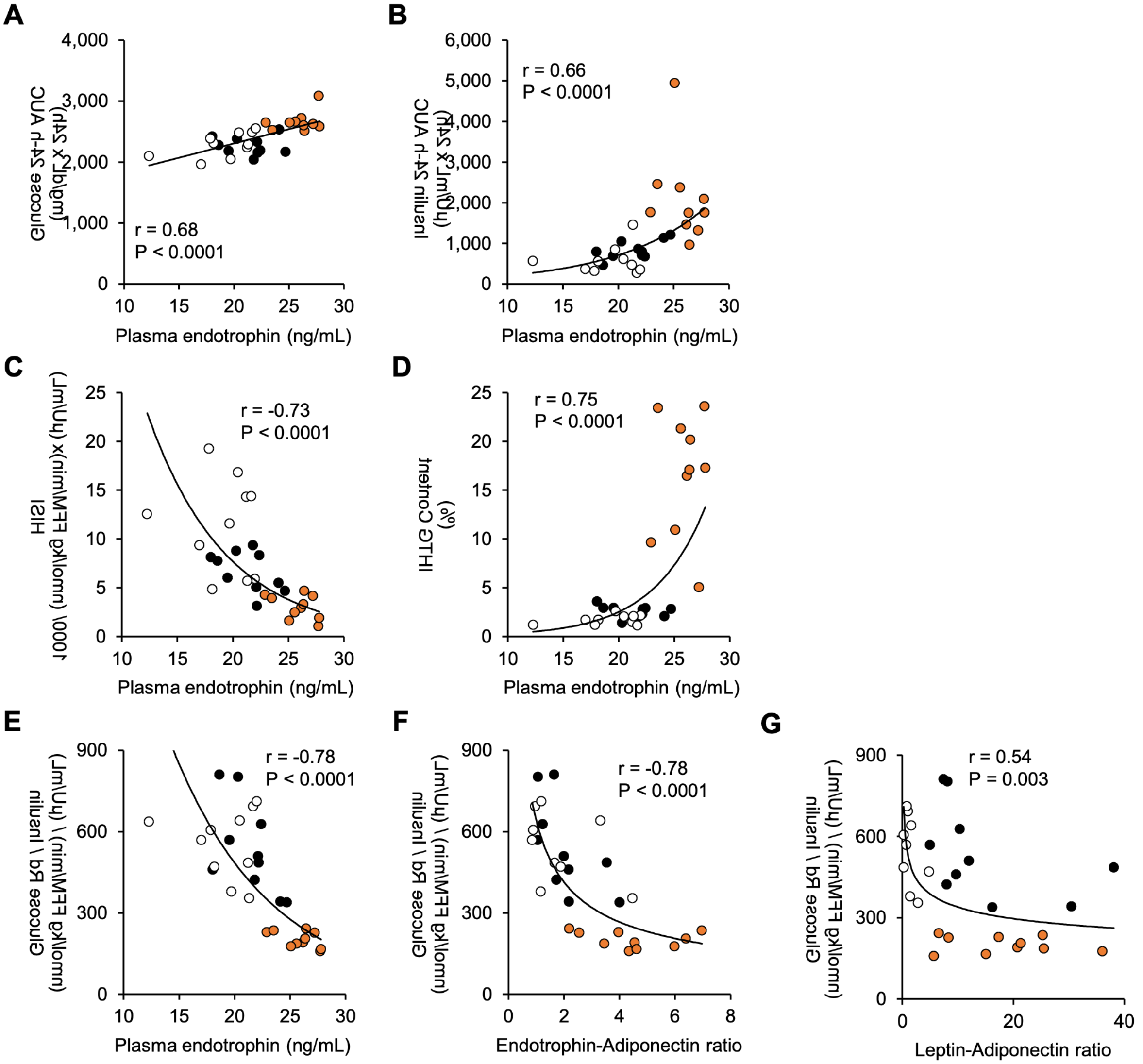


**Supplemental Figure 1. Relationships among plasma endotrophin concentration and serial 24-h plasma glucose and insulin concentrations and insulin sensitivity. (A-E)** Relationship between plasma endotrophin concentration and 24-h plasma glucose **(A)** and insulin **(B)** concentration areas-under-the curve (AUC), hepatic insulin sensitivity index (HISI) **(C)**, intrahepatic triglyceride (IHTG) content **(D)** and whole-body insulin sensitivity (glucose rate of disappearance (Rd) divided by plasma insulin concentration during a hyperinsulinemic-euglycemic clamp procedure) **(E)**. **(F-G)** Relationship between whole-body insulin sensitivity and plasma endotrophin-to-adiponectin ratio **(F)** and plasma leptin-adiponectin ratio **(G)** White, black, and orange circles represent participants in the Lean-IS, Obese-IS, and Obese-IR groups, respectively. All group sizes are n=10 Lean-IS, 10 Obese-IS and 10 Obese-IR except panels F and G, which are n=9 Lean-IS, 10 Obese-IS and 10 Obese-IR. Relationships among outcomes were evaluated by using linear and nonlinear regression analysis with the best fit to the data reported.

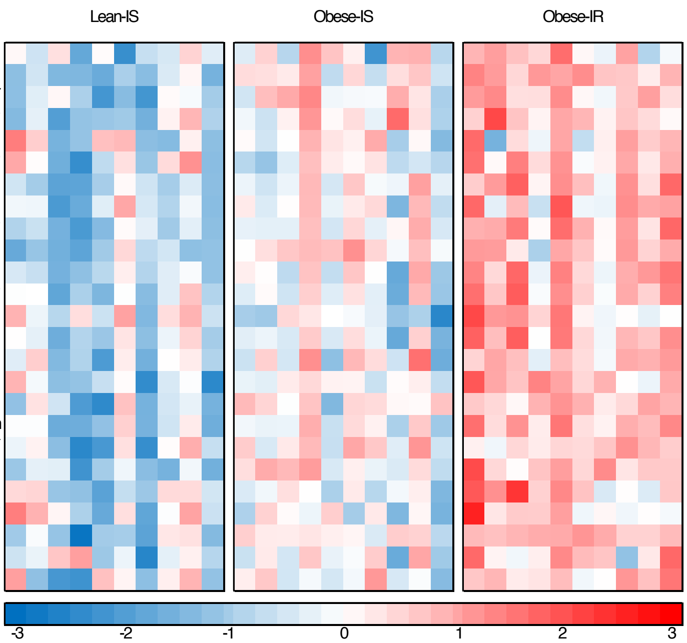


| | ANOVA P value | Association with plasma endotrophin r value | Association with plasma endotrophin P value |
|---|---|---|---|
| *HIF1A* | 0.073 | 0.39 | 0.035 |
| *CD86* | <0.001 | 0.79 | <0.001 |
| *TNF* | <0.001 | 0.72 | <0.001 |
| *CCL2* | <0.001 | 0.63 | <0.001 |
| *COL1A1* | 0.345 | 0.22 | 0.248 |
| *COL1A2* | 0.012 | 0.52 | 0.004 |
| *COL3A1* | <001 | 0.62 | <0.001 |
| *COL5A1* | 0.009 | 0.40 | 0.030 |
| *COL5A2* | <001 | 0.67 | <0.001 |
| *COL5A3* | <001 | 0.45 | 0.012 |
| *COL6A1* | <0.001 | 0.63 | <0.001 |
| *COL6A2* | <0.001 | 0.65 | <0.001 |
| *COL6A3* | 0.010 | 0.52 | 0.003 |
| Mean *COL6* | <0.001 | 0.63 | <0.001 |
| COL12A1 | 0.014 | 0.63 | <0.001 |
| COL14A1 | <001 | 0.71 | <0.001 |
| COL24A1 | <001 | 0.66 | <0.001 |
| Composite collagen expression | <0.001 | 0.68 | <0.001 |
| *LOX* | 0.006 | 0.58 | <0.001 |
| *TGFB1* | <0.001 | 0.65 | <0.001 |
| *FN1* | 0.003 | 0.71 | <0.001 |
| *MMP2* | 0.136 | 0.47 | 0.008 |
| *MMP9* | <0.001 | 0.63 | <0.001 |
| *MMP14* | 0.078 | 0.62 | <0.001 |
| *MMP16* | 0.026 | 0.26 | 0.172 |

**Supplemental Figure 2. Subcutaneous abdominal adipose tissue transcriptomics.** Heatmap representing gene expression of *HF1A*, markers of inflammation, individual collagen isoforms and composite collagen gene expression and key regulators of extracellular matrix remodeling and endotrophin production in Lean-IS (n=10), Obese-IS (n=10) and Obese-IR (n=10) groups. The data were normalized by calculating $\log_2$-transformed counts per million reads for each gene followed by Z-score transformation using the mean and standard deviation of all values for each gene. Composite collagen gene expression values were calculated as the average expression of 12 collagen isoforms (*COL1A1*, *COL1A2*, *COL3A1*, *COL5A1*, *COL5A2*, *COL5A3*, *COL6A1*, *COL6A2*, *COL6A3*, *COL12A1*, *COL14A1*, and *COL24A1*). Red indicates higher relative abundance, and blue indicates lower relative abundance. One-way ANOVA was used to compare gene expression between groups. Relationships between plasma endotrophin concentration and gene expression outcomes were evaluated by using linear and nonlinear regression analysis with the best fit to the data reported.

**SUPPLEMENTAL REFERENCES**


1. Yoshino M, Kayser BD, Yoshino J, Stein RI, Reeds D, Eagon JC, et al. Effects of Diet versus Gastric Bypass on Metabolic Function in Diabetes. *N Engl J Med.* 2020;383(8):721-32.

2. Petersen MC, Smith GI, Palacios HH, Farabi SS, Yoshino M, Yoshino J, et al. Cardiometabolic characteristics of people with metabolically healthy and unhealthy obesity. *Cell Metab.* 2024;36(4):745-61 e5.

3. Lean ME, Leslie WS, Barnes AC, Brosnahan N, Thom G, McCombie L, et al. Primary care-led weight management for remission of type 2 diabetes (DiRECT): an open-label, cluster-randomised trial. *Lancet.* 2018;391(10120):541-51.

4. Frimel TN, Deivanayagam S, Bashir A, O'Connor R, and Klein S. Assessment of intrahepatic triglyceride content using magnetic resonance spectroscopy. *J Cardiometab Syndr.* 2007;2(2):136-8.

5. Mifflin MD, St Jeor ST, Hill LA, Scott BJ, Daugherty SA, and Koh YO. A new predictive equation for resting energy expenditure in healthy individuals. *Am J Clin Nutr.* 1990;51(2):241-7.

6. Klein S, Fontana L, Young VL, Coggan AR, Kilo C, Patterson BW, et al. Absence of an effect of liposuction on insulin action and risk factors for coronary heart disease. *N Engl J Med.* 2004;350(25):2549-57.

7. Sun K, Park J, Gupta OT, Holland WL, Auerbach P, Zhang N, et al. Endotrophin triggers adipose tissue fibrosis and metabolic dysfunction. *Nat Commun.* 2014;5:3485.

8. Oh J, Kim CS, Kim M, Jo W, Sung YH, and Park J. Type VI collagen and its cleavage product, endotrophin, cooperatively regulate the adipogenic and lipolytic capacity of adipocytes. *Metabolism.* 2021;114:154430.

9. DeFronzo RA, Gunnarsson R, Bjorkman O, Olsson M, and Wahren J. Effects of insulin on peripheral and splanchnic glucose metabolism in noninsulin-dependent (type II) diabetes mellitus. *J Clin Invest.* 1985;76(1):149-55.

10. Koh HE, van Vliet S, Meyer GA, Laforest R, Gropler RJ, Klein S, et al. Heterogeneity in insulin-stimulated glucose uptake among different muscle groups in healthy lean people and people with obesity. *Diabetologia.* 2021;64(5):1158-68.

11. Baron AD, Brechtel G, Wallace P, and Edelman SV. Rates and tissue sites of non-insulin- and insulin-mediated glucose uptake in humans. *Am J Physiol.* 1988;255(6 Pt 1):E769-74.

12. Flannery AH, Bu D, Botkins M, Gianella F, Zhang N, An Z, et al. Endotrophin as a Biomarker for Severe Acute Kidney Injury and Major Adverse Kidney Events. *Kidney360.* 2024;5(8):1087-93.

13. Allison DB, Paultre F, Maggio C, Mezzitis N, and Pi-Sunyer FX. The use of areas under curves in diabetes research. *Diabetes Care.* 1995;18(2):245-50.

14. Mittendorfer B, Horowitz JF, and Klein S. Gender differences in lipid and glucose kinetics during short-term fasting. *Am J Physiol Endocrinol Metab.* 2001;281(6):E1333-9.

15. Korenblat KM, Fabbrini E, Mohammed BS, and Klein S. Liver, muscle, and adipose tissue insulin action is directly related to intrahepatic triglyceride content in obese subjects. *Gastroenterology.* 2008;134(5):1369-75.

16. Yamaguchi S, Franczyk MP, Chondronikola M, Qi N, Gunawardana SC, Stromsdorfer KL, et al. Adipose tissue NAD(+) biosynthesis is required for regulating adaptive thermogenesis and whole-body energy homeostasis in mice. *Proc Natl Acad Sci U S A.* 2019;116(47):23822-8.

17. Beals JW, Smith GI, Shankaran M, Fuchs A, Schweitzer GG, Yoshino J, et al. Increased Adipose Tissue Fibrogenesis, Not Impaired Expandability, Is Associated With Nonalcoholic Fatty Liver Disease. *Hepatology.* 2021;74(3):1287-99.
